# An *ab initio* study of the rovibronic spectrum of sulphur monoxide (SO): diabatic *vs.* adiabatic representation†

**DOI:** 10.1039/d2cp03051a

**Published:** 2022-09-23

**Authors:** R. P. Brady, S. N. Yurchenko, G.-S. Kim, W. Somogyi, J. Tennyson

**Affiliations:** Department of Physics and Astronomy, University College London Gower Street WC1E 6BT London UK s.yurchenko@ucl.ac.uk; Dharma College, Dongguk University 30, Pildong-ro 1-gil Jung-gu Seoul 04620 Korea

## Abstract

We present an *ab initio* study of the rovibronic spectra of sulphur monoxide (^32^S^16^O) using internally contracted multireference configuration interaction (ic-MRCI) method and aug-cc-pV5Z basis sets. It covers 13 electronic states X^3^Σ^−^, a^1^Δ, b^1^Σ^+^, c^1^Σ^−^, A′′^3^Σ^+^, A′^3^Δ, A^3^Π, B^3^Σ^−^, C^3^Π, d^1^Π, e^1^Π, C′^3^Π, and (3)^1^Π ranging up to 66 800 cm^−1^. The *ab initio* spectroscopic model includes 13 potential energy curves, 23 dipole and transition dipole moment curves, 23 spin–orbit curves, and 14 electronic angular momentum curves. A diabatic representation is built by removing the avoided crossings between the spatially degenerate pairs C^3^Π–C′^3^Π and e^1^Π–(3)^1^Π through a property-based diabatisation method. We also present non-adiabatic couplings and diabatic couplings for these avoided crossing systems. All phases for our coupling curves are defined, and consistent, providing the first fully reproducible spectroscopic model of SO covering the wavelength range longer than 147 nm. Finally, an *ab initio* rovibronic spectrum of SO is computed.

## Abstract 2

We present an *ab initio* study of the rovibronic spectra of sulphur monoxide (^32^S^16^O) using internally contracted multireference configuration interaction (ic-MRCI) method and aug-cc-pV5Z basis sets where non-adiabatic effects are treated.

## Graphical abstract

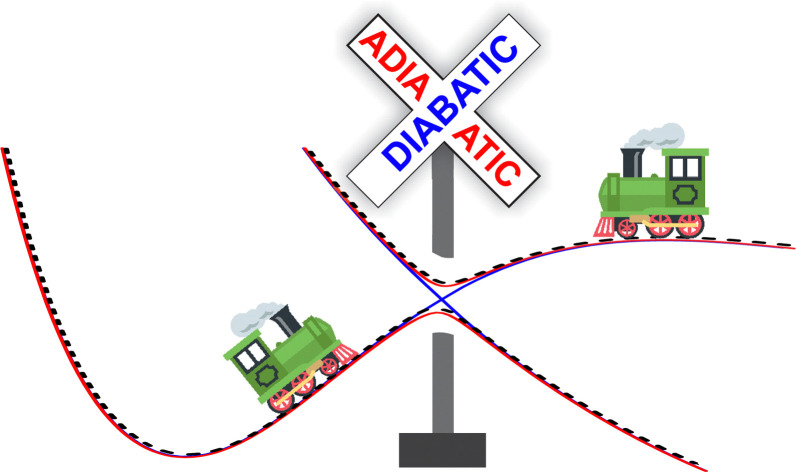

## 1 Introduction

Sulphur monoxide (SO) has many experimental and observational applications, making it a molecule of continuing interest in spectroscopic and kinetic studies. SO is an important intermediate in the oxidation of sulphur compounds within combustion reactions,^1^ making SO studies applicable to environmental chemistry since sulphur oxides lead to the acidification/pollution of Earth's atmosphere.^2–5^ Burkholder *et al.*^6^ show the importance of SO within chemical reactions of Earth's atmosphere due to its high reactivity and chemical involvement with N_2_ and O_2_. Additionally, UV lasing in SO was demonstrated through optically pumping transitions within the B^3^Σ^−^–X^3^Σ^−^ electronic band^7^ and then later for UV energy storage lasers.^8^ SO also has astrophysical importance, its Zeeman splitting has been used to probe the magnetic fields of dense star-forming regions, such as the Orion molecular cloud, for field strengths ≥10^−2^ G^9,10^ and its presence within warm chemistry's mean it is an excellent shock tracer.^11,12^

The frontier orbitals of SO resemble that of carbon monoxide where the two π* valence electrons mean SO has a X^3^Σ^−^ ground state similar to O_2_ and S_2_. Being isoelectronic with O_2_, SO has two low-lying metastable a^1^Δ and b^1^Σ^+^ states which are relatively short lived due to large spin–orbit coupling. SO has been subject to pure rotational,^13–15^ electronic,^16–20^ and ro-vibrational^21–23^ experimental spectroscopic studies. More recently, Bernath *et al.*^24,25^ produced empirical line lists for the b^1^Σ^+^–X^3^Σ^−^ and a^1^Δ–X^3^Σ^−^ rovibronic bands and for the X^3^Σ^−^ and a^1^Δ rovibrational bands of SO. The literature also contains many theoretical studies of SO, the most comprehensive, and one we compare to often, being Yu and Bian^26^ who give spectroscopic constants on all electronic states we consider here (except (3)^1^Π) computed through internally contracted multireference configuration interaction (ic-MRCI) methods using aug-cc-pV5Z basis sets. Another important theoretical work is by Sarka and Nanbu^27^ who study the UV region of SO non-adiabatically where they compute PECs, DMCs, and non-adiabatic couplings (NACs) for the X^3^Σ^−^, A^3^Π, B^3^Σ^−^, C^3^Π, C′^3^Π, (3)^3^Σ^−^, (4)^3^Π, and (5)^3^Π states at a MRCI-F12+Q level of theory using aug-cc-pV(5+d)Z basis sets. Sarka and Nanbu^27^ are also the first to compute cross-sections for SO longer than 190 nm in the UV.

Despite the strong experimental, theoretical, and observational baseline for SO, to this date the existing spectroscopic line list data for SO are limited in coverage. HITRAN^28^ contains only transitions between electronic states X^3^Σ^−^, a^1^Δ and b^1^Σ^+^ considering relatively low vibrational excitations. SO data and spectroscopic databases NIST^29^ and CDMS^30^ contain data covering the microwave region only. In this study we aim to provide a strong theoretical base from which an accurate spectroscopic description of SO with large coverage can be made through the refinement of our *ab initio* model to empirically determined energies in a future work. To achieve this we calculate *ab initio* potential energy curves (PECs), spin–orbit curves (SOCs), electronic angular momentum curves (EAMCs), and electric (transition) dipole moment curves ((T)DMCs) for the 13 lowest electronic states of SO (X^3^Σ^−^, a^1^Δ, b^1^Σ^+^, c^1^Σ^−^, A′^3^Δ, A′′^3^Σ^+^, A^3^Π, B^3^Σ^−^, C^3^Π, d^1^Π, e^1^Π, C′^3^Π, (3)^1^Π) at an MRCI level of theory using aug-cc-pV5Z basis sets. The relative phases of the the non-diagonal couplings and transition dipole moments provided are fully self-consistent,^31^ which is crucial for reproducible spectroscopic studies.

Our *ab initio* curves of SO are adiabatic as computed under the Born–Oppenheimer approximation^32^ and so the spatially degenerate states e^1^Π, (3)^1^Π and C^3^Π, C′^3^Π of SO exhibit avoided crossings due to their shared symmetries, where the non-adiabatic effects play important role. The associated non-adiabatic couplings (NACs) arise from the nuclear motion kinetic energy operator acting on electronic wavefunctions and contain first- and second-order differential operators with respect to the nuclear coordinates. Inclusion of NACs within numerical dynamics calculations are computationally costly when using analytical forms for the PECs and couplings because of both the cusp-like nature of PECs and the NACs having singularities at the region of spatial degeneracy.^33–36^ This makes the fitting of analytical forms to the PECs and couplings almost impossible. Here we explore a diabatisation procedure to transform the adiabatic basis to the diabatic basis using NACs reconstructed from PECs as opposed to obtaining them *ab initio*,^27^ where the adiabatic first-order differential couplings are negligible or vanish, at the cost of inducing off-diagonal potential energy couplings,^37^ or diabatic couplings (DCs).^33,34^ In this representation, the electronic wavefunctions are smooth which lessens both numerical problems encountered in calculations within the adiabatic representation and the computational cost in computing NACs. Diabatisation helps recover non-Born–Oppenheimer dynamics^34,38^ and is important for processes such as collisions between open-shell molecules with spatially degenerate electronic states.^35,39–42^ The importance of the non-adiabatic effects on the spectral properties of SO is analysed.

## 2 Computational details

**Fig. 1:**
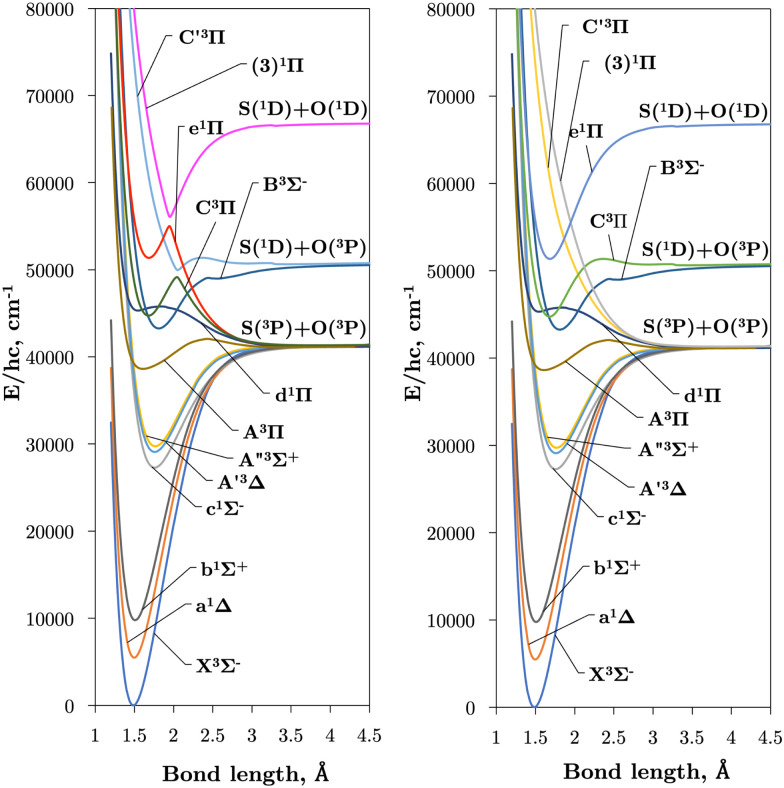
Plots of our 13 *ab initio* PECs covering the first 7 triplet and 6 singlet electronic states up to 80 000 cm^−1^ over inter-nuclear separations 1.0–4.5 Å. The adiabatic and diabatic representations of the PECs are shown in the left and right hand panels respectively.

**Fig. 2:**
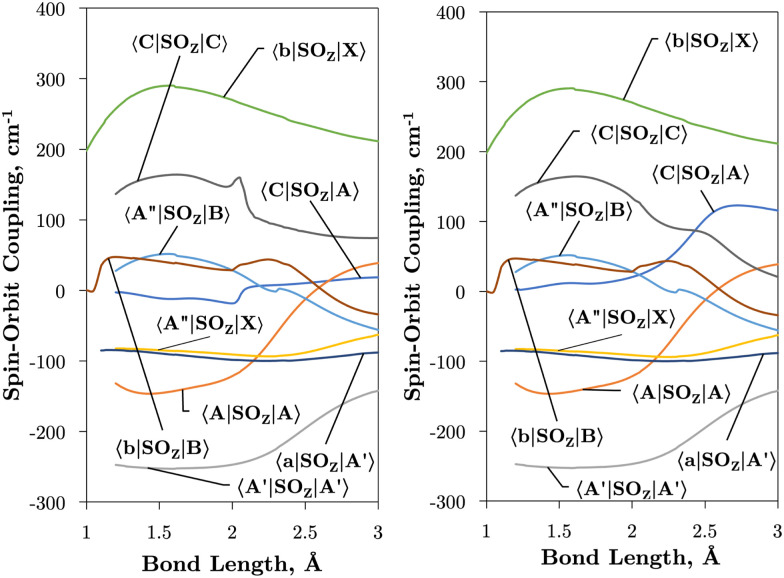
*Ab initio* spin–orbit curves between states of the same *Λ* (projection of the angular momentum) as a function of bond length. In general, the spin–orbit coupling is strong also between states of different multiplicity. These curves have been multiplied by −*i* for visual purposes.

**Fig. 3:**
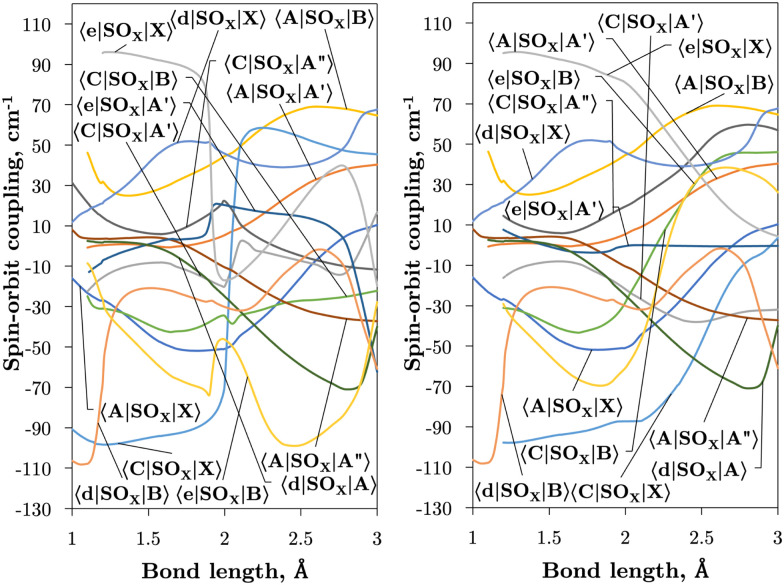
*Ab initio* spin–orbit MOLRPO matrix elements in the adiabatic (left) and diabatic (right) representations between states of different values of *Λ* as a function of bond length. These curves have been multiplied by −*i* for visual purposes.

**Fig. 4:**
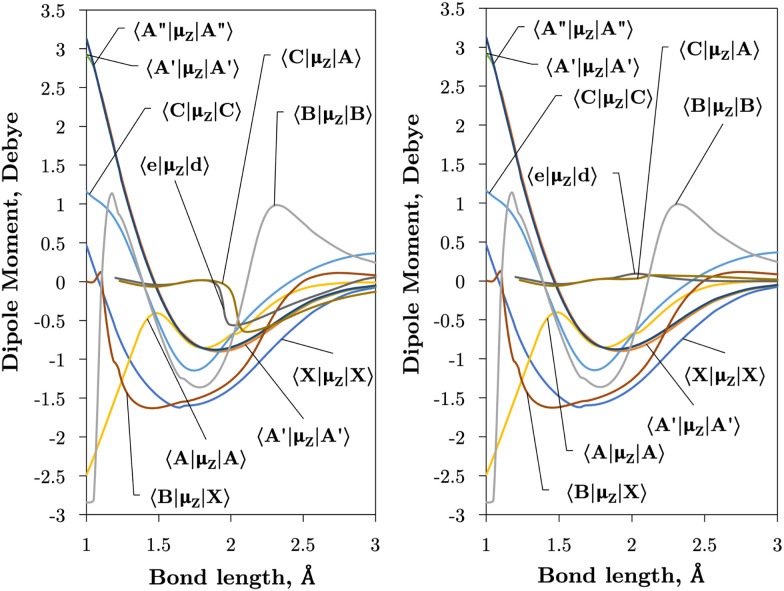
*Ab initio* (transition) dipole moment matrix elements (Debye) between states of the same symmetry (*Λ* and multiplicity) in the adiabatic (left) and diabatic (right) representations as a function of the bond length (Å).

**Fig. 5:**
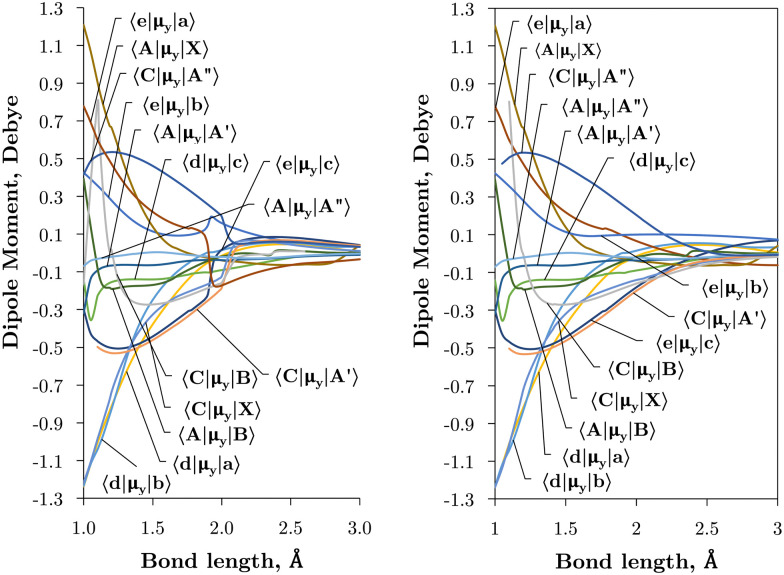
*Ab initio* transition dipole moment curves (Debye) between states of different symmetry in the adiabatic (left) and diabatic (right) representations as a function of the bond length (Å).

**Fig. 6:**
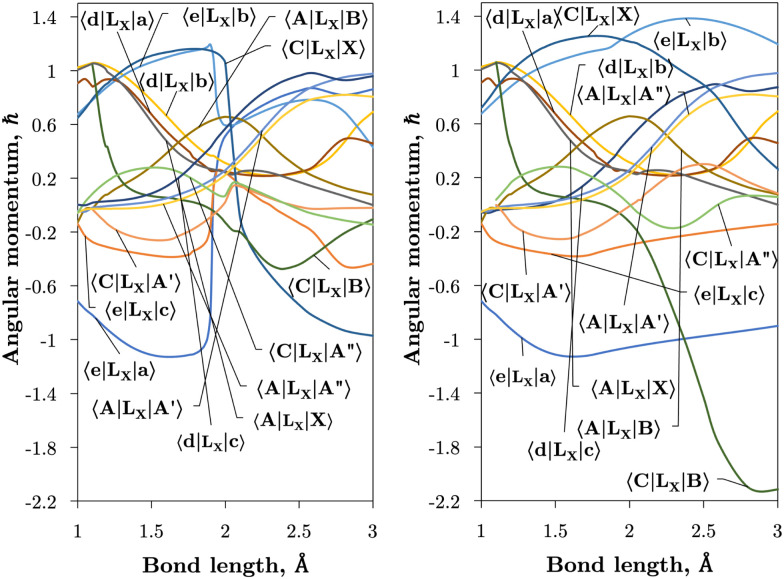
*Ab initio* electronic angular momentum curves in the adiabatic (left) and diabatic (right) representation plotted over bond length. *L*_*x*_ means the *x*-component of the electronic angular momentum. These curves have been multiplied by (−*i*) for visual purposes.

Internally-contracted multi-reference configuration interaction (icMRCI) *ab initio* calculations for the 13 lowest states of SO correlating with S(^3^P) + O(^3^P), S(^1^D) + O(^3^P) and S(^1^D) + O(^1^D) were performed using Molpro^43^ with aug-cc-pV5Z basis sets,^44,45^ using molecular orbitals obtained from state-averaged complete active space self-consistent field (CASSCF) calculations. Under *C*_2V_ point group symmetry all *ab initio* calculations started with 14 (8σ, 3π_*x*_, 3π_*y*_) orbitals which included 6 closed (4σ, 1π_*x*_, 1π_*y*_) orbitals. The active space occupying 12 active electrons consisted of 8 (5σ–8σ, 2π, 3π) valence orbitals. The PECs, including the 8 bound states X^3^Σ^−^, a^1^Δ, b^1^Σ^+^, c^1^Σ^−^, A′′^3^Σ^+^, A′^3^Δ, A^3^Π, and B^3^Σ^−^ are shown in Fig. 1, as well as the adiabatic e^1^Π–(3)^1^Π and C^3^Π–C′^3^Π systems. The EAMC, SOCs (both diagonal and non-diagonal), DMCs (diagonal and transition) computed and used later for cross-section calculations are shown in Fig. 2–6 in the original adiabatic representation as computed by Molpro 2015^43^ (left) and the diabatic representation (right). Further discussion of the diabatisation is given in the next section.

## 3 Diabatisation

### 3.1 Non-adiabatic couplings (NACs)

The commonly employed Born–Oppenheimer approximation assumes the nuclear dynamics evolve adiabatically on a single, uncoupled electronic potential energy surface. This results in the appearance of avoided crossings between electronic states that are degenerate in energy at some geometry and large gradients in couplings connecting these states. The Born–Huang expansion^46^ allows one to recover the non-adiabatic behaviour in the region of the avoided crossing by including non-adiabatic couplings (NACs) which introduce off-diagonal kinetic energy terms in the molecular Hamiltonian. An alternative approach is to transform the electronic Hamiltonian to the diabatic basis, where electronic states are coupled *via* off-diagonal potential energy terms, termed diabatic couplings (DCs).^33,34,37^ The transformation from the adiabatic to the diabatic basis is described by a unitary matrix *U*, which is parametrically dependent on the NAC term,1
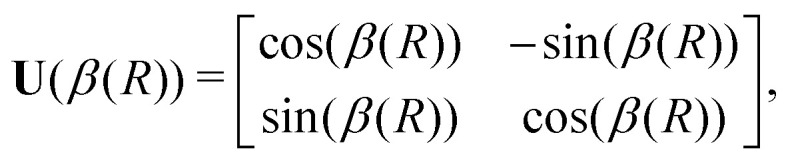
where the mixing angle *β*(*R*) is obtained by integrating the functional form of the non-adiabatic derivative coupling 
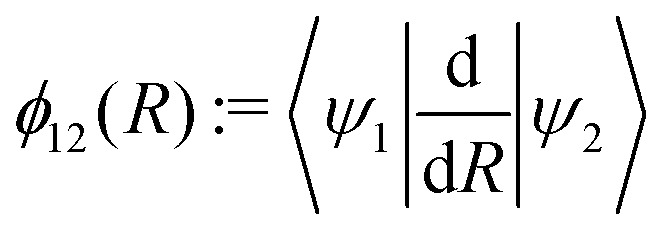
^35,47–49^2
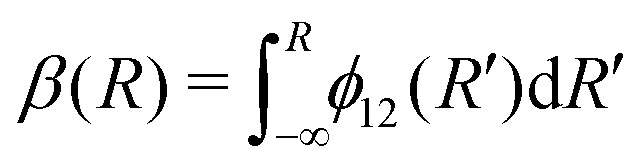
with |*ψ*_1_〉 and |*ψ*_2_〉 as the lower and upper energy electronic wavefunctions in the adiabatic representation.

Writing the two-state electronic Hamiltonian in terms of the adiabatic potential energy curves *V*^a^_1_(*R*) and *V*^a^_2_(*R*),3
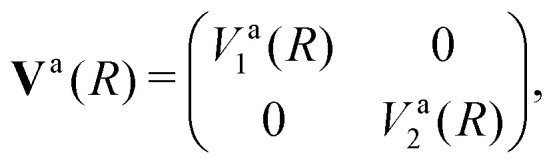
we can obtain the diabatic Hamiltonian by applying the unitary transformation ***U***(*β*(*R*)),4
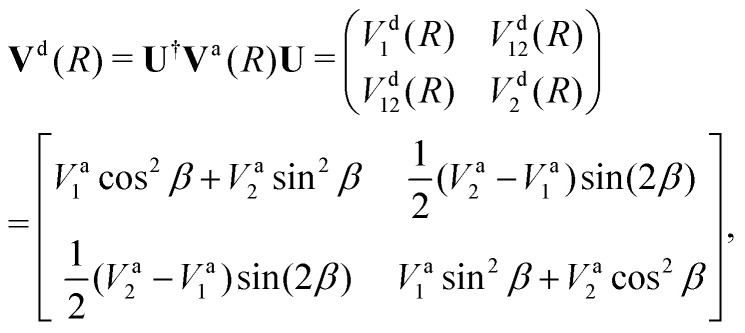
where the superscripts ‘d’ and ‘a’ refer to the diabatic and adiabatic bases, respectively, and the off-diagonal elements *V*^d^_12_(*R*) are the DCs. The reverse transformation is obtained by diagonalising the diabatic representation of the electronic Hamiltonian.

**Fig. 7:**
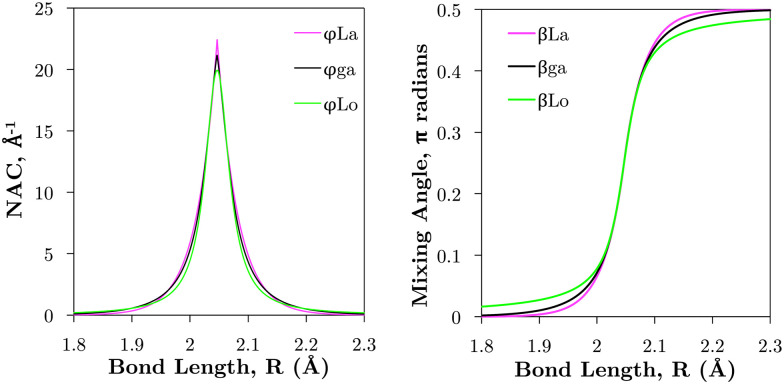
Comparison of example NACs (*ϕ*_*ij*_) and corresponding diabatic mixing angles (*β*) between the Lorentzian (‘Lo’), Laplacian (‘La’), and geometrically averaged (‘ga’) cases as described in the text. These curves are computed for the C^3^Π and C′^3^Π non-adiabatic interaction (see Section 3.2.1 and Fig. 1).

The NAC can be computed *via* quantum-chemistry methods from the electronic wavefunctions, as done by Sarka and Nanbu^27^ for SO. It is symmetrical with a cusp at the crossing point *R*_c_. Alternatively, the NAC curves are modelled using, *e.g.* a Lorentzian,^50^ as given by5
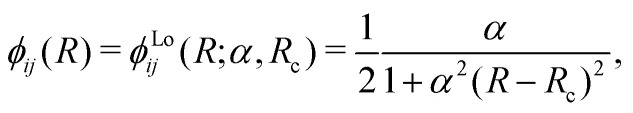
where *α* is the inverse half-width-at-half-maximum (HWHM), or a Laplacian6
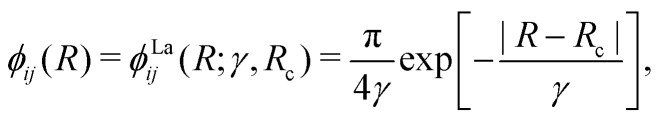
where *γ* is a damping constant related to the HWHM, superscripts ‘Lo’ and ‘La’ mean Lorentzian and Laplacian respectively, and the normalisation 
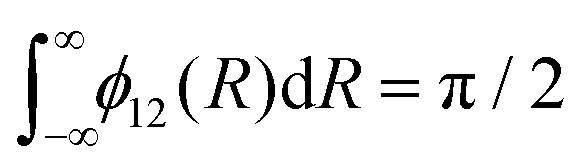
 is applied. Fig. 7 illustrates the C^3^Π–C′^3^Π NAC modelled in this work using a Lorentzian and Laplacian function. The mixing angle *β*(*R*), determined through eqn (2), ranges from 0 to π/2 going through π/4 at the crossing point *R* = *R*_c_ can also be seen in Fig. 7.

The Lorentzian was shown to provide a good description of the *ab initio* NACs around the crossing point^48,49,51,52^ (see Fig. 7) but diverges at large distances *R* from *R*_c_ causing improper shaped diabatic PECs by decaying too slowly.^48,49,51,53^ It has been discussed that some damping functions can be introduced to correct the Lorentzian's slow decay using properties such as dipole moments, but determination of their fitting parameters is both difficult and requires extra calculations.^50,51^ Laplacians underestimate NACs far in the wings and overestimate them near the crossing point.^48^ One can show that NACs have an overlap dependence on the internuclear separation,^51,54^*R*, which is given properly by a Laplacian.^48^ The undesirable features of these NAC models can be mitigated by their combination^48,49,51,53^ to which we base our diabatisation procedure on. Our method of augmenting the Lorentzian with a Laplacian is discussed in Section 3.2.

### 3.2 Diabatisation

Here we explore the so-called ‘property based diabatisation’ method^35^ and construct diabatic potentials using the condition of having no avoiding crossing, which we define as to minimise their second derivatives in the vicinity of the crossing point *R*_c_:7
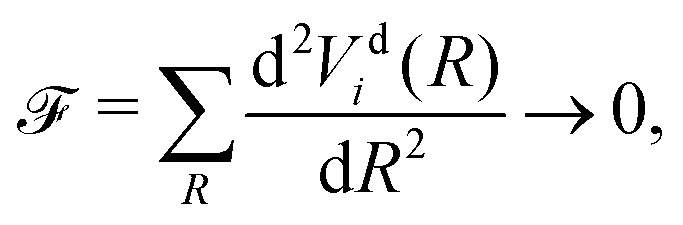
hence creating the smoothest PECs *V*^d^_1_(*R*) and *V*^d^_2_(*R*).

In order to mitigate the undesirable properties of the Lorentzian and Laplacian functional forms, we follow the approach by An and Baeck^48^ and represent the mixing angle *β* by the following combination of the mixing angles determined from the Lorentzian and Laplacian NACs in eqn (2), *β*^Lo^(*R*) and *β*^La^(*R*)8
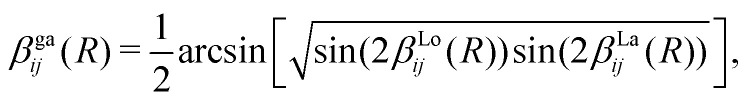
where the ‘ga’ superscript refers to the geometrically averaged diabatic mixing angle (see Fig. 7). Eqn (8) must not be taken as the geometric average of *β*^Lo^ and *β*^La^, but rather originates from the simple geometric average of *V*^Lo^_12_ and *V*^La^_12_ (see An and Baeck^48^ and Appendix). An and Baeck^48^ also showed that an optimal relation between the parameters *α* and *γ* exists which given by

9 *α* × *γ* = 1.397

providing maximal overlap between the Lorentzian and Laplacian functions over the bond length.

**Fig. 8:**
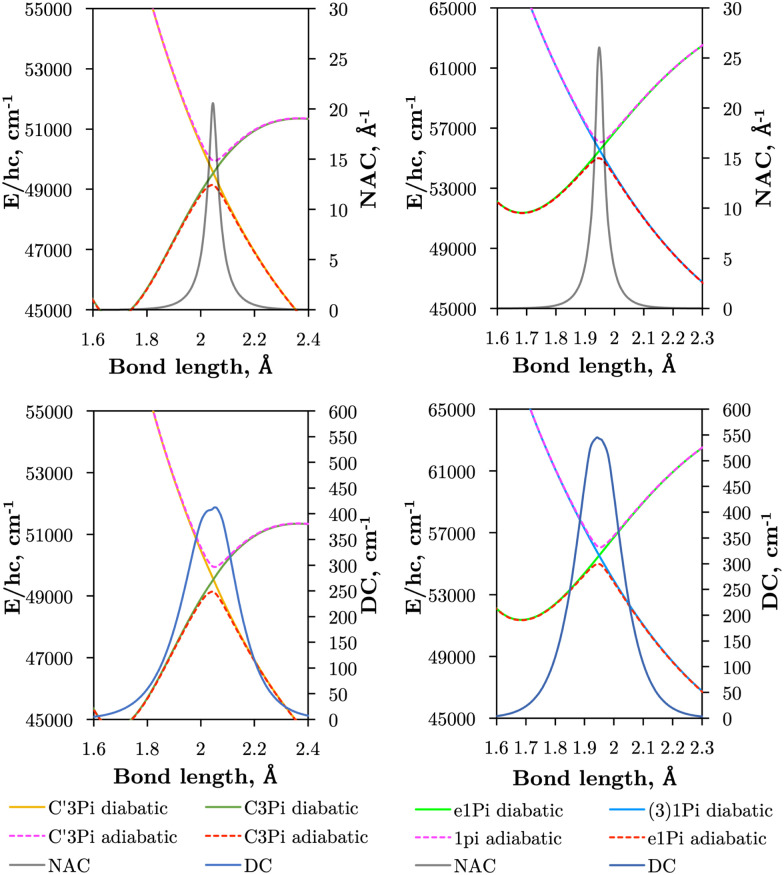
Illustration of the avoided crossings between e^1^Π–(3)^1^Π and (right panels) and C^3^Π–C′^3^Π states of SO (left panels) are shown, where adiabatic PECs are presented as dashed lines and diabatic ones in solid lines. Superimposed are the DCs (*V*^ga^_12_, bottom panels), and NACs *(ϕ*^ga^_*ij*_, top panels).

Where our method diverges from that of An and Baeck^48^ is in the determination of the crossing point *R*_c_ and the Lorentzian parameter *α*. An and Baeck^48^ obtained *R*_c_ and *α* through fitting a Lorentzian to a NAC computed with Molpro. Instead, we determine a set of parameters {*R*_c_,*α*} by fulfilling the condition given in eqn (7), to which the Laplacian parameter *γ* is instantly obtained through eqn (9). Using the theory developed in Section 3.1 and eqn (8) the diabatising transformation ***U***^ga^ corresponding to the ‘geometrically averaged’ NAC is found. With this the diabatic potential energies and DC elements can be obtained through the simple matrix transformation in eqn (4). The diabatic PECs for SO can be seen in Fig. 1 and a closeup of the avoided crossings between the e^1^Π–(3)^1^Π and C^3^Π–C′^3^Π states of SO superimposed with their DCs, *V*^ga^_*ij*_, and NACs, *ϕ*^ga^_*ij*_, are illustrated in Fig. 8. Fig. 8 shows that the pair of singlet states are coupled more strongly than the triplet states, and also reveals the DCs to be slightly asymmetric. This is to be expected since the DCs depend on the difference *V*^a^_2_–*V*^a^_1_ which can be asymmetrical in nature. We see an effect of this especially in the DC between the triplet states where the adiabatic PEC turning points are offset to each other by ∼0.01 Å.

#### 3.2.1 Potential energy curves

Fig. 1 presents *ab initio* PECs of the 13 lowest energy electronic states of SO. The C^3^Π exhibits an avoided crossing at *R* ∼ 2.05 Å due to a non-adiabatic coupling with the dissociative C′^3^Π, which lends a dissociative character to the C^3^Π in the long-range region. Similarly, the e^1^Π state exhibits an avoided crossing at 1.95 Å, due to the singlet state (3)^1^Π.^26^ These non-adiabatic interactions produce large gradients in coupling curves connecting these states within the region of the avoided crossing, as shown for EAMCs, SOC, and DMCs in Fig. 2–6. The equilibrium geometry of the C^3^Π state also lies very close in energy to those of the d^1^Π and B^3^Σ^−^ states, and so we can expect perturbations in the energy levels around their minima which was reported and confirmed experimentally by Liu *et al.*^55^ It is worth noting that the A′^3^Δ and A′′^3^Σ^+^ states lie very close across the range of nuclear geometries included in these calculations, but do not cross.

Table 1 provides values for the optimized NAC parameters *α*,*γ*,*R*_c_ used to diabatise the energy degenerate pairs, which are visualised in Fig. 8. We see that the effect of diabatisation is to smooth the PECs, as enforced by eqn (7) with no avoided-crossing. The non-Born–Oppenheimer dynamics, initially manifested in the nuclear kintetic energy, has been rotated into the potential and coupling terms connecting the non-adiabatically interacting states. We now produce PECs that can be easily modelled by analytical forms in the diabatic representation, allowing us to tune them to experimental data in a future study where we aim to produce an empirically accurate line list.

**Table 1:** Optimised parameters *α* (inverse Lorentzian HWHM), *γ* (Laplacian damping parameter), and *R*_c_ (avoided crossing/centroid position) for the Lorentzian and Laplacian NACs used to diabatise the e^1^Π and C^3^Π state PECs (see Fig. 1)

State	*α*	*γ*	*R*_c_ (Å)
e^1^Π	52.422	0.027	1.949
C^3^Π	39.859	0.035	2.047

Table 2 compares the equilibrium potential energies, *T*_e_ (cm^−1^), equilibrium bond lengths, *R*_e_ (Å), and dissociation energies, *D*_e_ (eV), of the 11 lowest singlet and triplet states of SO determined directly from the *ab initio* adiabatic PECs presented in Fig. 1 to both calculations^26,27,56–58^ and experiment.^24,57,59–61^ Our bond lengths show good agreement to both theoretical and empirical values, with better agreement to experiment than previous calculations for the b^1^Σ^+^, A^3^Π, and d^1^Π states. Worse agreements are seen for our *T*_e_ values, the most accurate being *T*_e_(A^3^Π), *T*_e_(C^3^Π), and *T*_e_(e^1^Π) within 152 cm^−1^, 210 cm^−1^, and 338 cm^−1^ of experiment respectively. Lastly, we see worse agreements between our *ab initio* dissociation energies to both experiment and calculations, to which we underestimate. It is apparent that our dissociation asymptotes are the major source of inaccuracy in our *ab initio* SO model and probably arise because we do not include Sulfur specific diffuse functions in our basis set during *ab initio* calculations. A future goal will be to mitigate the undesirable PEC features by refining our *ab initio* model to experimental transition data, to which we will address when producing the final SO line list. We note that the reported Bernath *et al.*^24^*R*_e_ values in Table 2 were derived from the *B*_*ν*=0_ rotational constant, and show close agreements to within 0.006 cm^−1^ Å and 0.002 cm^−1^ Å to our bond lengths for the a^1^Δ and b^1^Σ^+^ respectively. Our fundamental vibrational energy of the X^3^Σ^−^ state is found approximately 30 cm^−1^ too high from the experiment, which is to be expected with MRCI calculations, and provides insight to the accuracy of the other computed states and couplings, which will also require empirical tuning.

**Table 2:** Comparison of *ab initio* values of the equilibrium potential energies *T*_e_ (cm^−1^), the dissociation energy *D*_e_ (eV) to the adiabatically correlated asymptotes, and equilibrium bond length *R*_e_ (Å) from this work to the values from the literature. Parameters next to bold state symbols correspond to the *ab initio* PECs calculated in this study. Calculations^26,56–58^ use the MRCI/cc-aug-pV5Z level of theory, calculation by Sarka and Nanbu^27^ use the MRCI-F12+Q/cc-aug-pV(5+d)Z level of theory, and experiments are photoion-photoelectron coincidence,^60^ multiphoton ionization^57^ and Ar + SO_2_ afterglow,^61^ spectroscopes as well as from the recent analysis by Bernath *et al.*^24^

State	*T*_e_ (cm^−1^)	*D*_e_ (eV)	*R*_e_ (Å)	State	*T*_e_ (cm^−1^)	*D*_e_ (eV)	*R*_e_ (Å)
X^3^Σ^−^	0	5.1253	1.4821	A′^3^Δ	29 097.8878	1.503	1.7571
Calc.^26^	0	5.418	1.4865	Calc.^26^	29 828	1.72	1.7649
Expt.^59^		5.429	1.481	B^3^Σ^−^	43 255.0097	0.9212	1.8121
Calc.^27^	0	5.475	1.4925	Calc.^27^	41 706.5886	1.4022	1.7868
Expt.^60^			1.481	Calc.^26^	41 314	1.387	1.782
Calc.^56^			1.481	Expt.^60^	41 629	1.410^62^	1.775
Calc.^58^			1.493	Calc.^58^	41 206		1.794
a^1^Δ	5479.8013	4.4486	1.4979	d^1^Π	45 309.0766	0.0587	1.545
Calc.^26^	5936	4.682	1.4945	Calc.^26^	44 166	0.189	1.5475
Expt.^59^	5873	4.647	1.4919	Expt.^57^	43 902	0.195	1.5303
Calc.^58^	5883		1.502	Calc.^58^	44 975	0.059	1.723
Expt.^24^			1.4920	Calc.^57^	44 471	0.14	1.553
b^1^Σ^+^	9774.1938	3.9154	1.5057	A′′^3^Σ^+^	29 731.2077	1.4417	1.765
Calc.^26^	10 548	4.112	1.5062	Calc.^26^	30 495	1.637	1.7701
Expt.^59^	10 510	4.137	1.5001	Expt.^61^	30 692		
Calc.^58^	10 576		1.514	Calc.^58^	30 025		1.776
Expt.^24^			1.5035	C^3^Π	44 719.2593	0.5489	1.6786
A^3^P	38 607.6737	0.4246	1.6079	Calc.^26^	44 033	0.609	1.6692
Calc.^27^	38 879.2948	0.6441	1.5946	Calc.^27^	44 909.0901	0.6027	1.6727
Calc.^26^	38 334	0.665	1.6196	Expt.^57^	44 381		1.654
Expt.^59^	38 455	0.662	1.609	Calc.^58^	44 038		1.681
Calc.^56^	38 880		1.613	e^1^Π	51 347.9346	0.4524	1.6864
Calc.^58^	38 931		1.719	Calc.^26^	51 224	0.45	1.6826
c^1^Σ^−^	27 274.9752	1.7679	1.7571	Expt.^57^	51 558		1.6774
Calc.^26^	28 544	1.879	1.7617	Calc.^58^	51 258		1.685

**Fig. 9:**
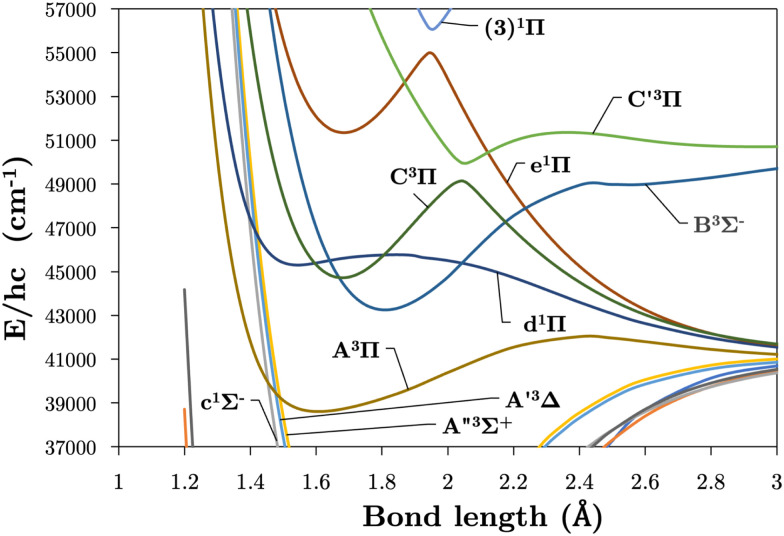
Our *ab initio* PECs in the region 37 000–57 000 cm^−1^ showcasing the various state crossings.

The intersections between states of different symmetries obtained in our calculations can be seen in Fig. 9. The d^1^Π and C^3^Π states cross at 1.59 and 1.82 Å, d^1^Π and B^3^Σ^−^ at 1.64 and 2.20 Å, both agreeing with Yu and Bian^26^ intersection locations of 1.62 and 1.80 Å and 1.60 and 2.14 Å, respectively. The intersection of the e^1^Π with the B^3^Σ^−^ state occurs at 2.4 Å in our calculations, somewhat larger than the value of 2.3 Å reported by Yu and Bian.^26^ Since the e^1^Π and d^1^Π states become repulsive at 1.92 Å and 1.9 Å respectively, crossings beyond these geometries provide potential predissociation pathways for the C^3^Π and B^3^Σ^−^ states. Yu and Bian^26^ also show that the C′^3^Π state crosses the B^3^Σ^−^ state at 2.25 Å, to which they state predissociation of B^3^Σ^−^ through this C′^3^Π state is possible. Sarka and Nanbu^27^ also give intersections *R*(C,B) = 1.57,2.21 Å as opposed to our values of *R*(C,B) = 1.67, 2.18 Å.

Lastly, we report further crossings between the c^1^Σ^−^, A′^3^Δ, and A′′^3^Σ^+^ states and the A^3^Π and d^1^Π states of SO at *R*(c,A) = 1.46 Å, *R*(A′,A) = 1.48 Å, *R*(A′′,A) = 1.50 Å, *R*(c,d) = 1.40 Å, *R*(A′,d) = 1.42 Å, and *R*(A′′,d) = 1.43 Å. These crossings agree with those reported by Yu and Bian^26^ between c^1^Σ^−^, A′^3^Δ, and A′′^3^Σ^+^ with A^3^Π in the region 1.47–1.51 Å and crossings between c^1^Σ^−^, A′^3^Δ, A′′^3^Σ^+^ with d^1^Π in the region 1.42–1.45 Å.

#### 3.2.2 Spin–orbit and electronic angular momentum curves

Fig. 6 shows the EAMCs of SO both in the adiabatic and diabatic representations, where their relative phases are carefully maintained according with their *ab initio* values.^31^ Without this, using couplings and any other non-diagonal properties in rovibronic calculations become meaningless. The phase of the EAMCs often changes after the crossing point, lending different long-range total angular momenta of the S + O atomic system important in dissociation mechanisms.

**Fig. 10:**
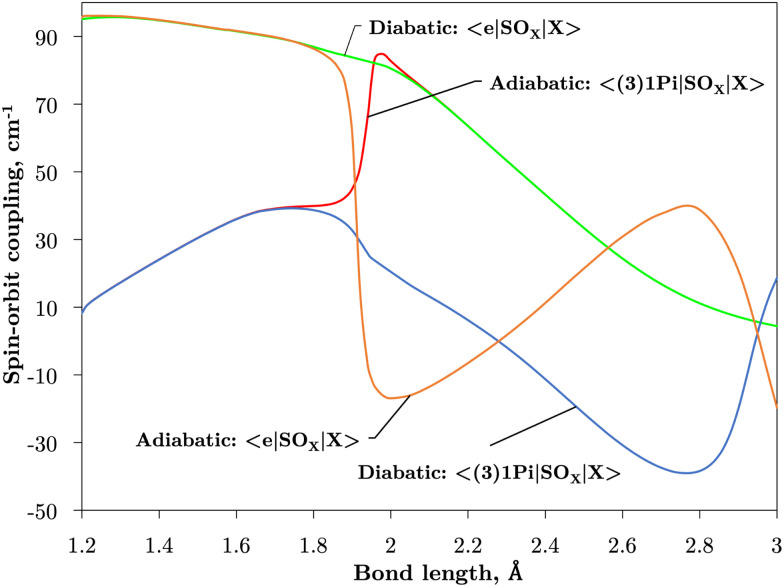
The diabatisation process for the curves 〈e^1^Π|SO_*x*_|X^3^Σ^−^〉 and 〈(3)^1^Π|SO_*x*_|X^3^Σ^−^〉, which become smooth as a result of the diabatisation. These curves have been multiplied by −*i* for visual purposes.

Fig. 2 and 3 plot the *z* (SO_*z*_) and *x* (SO_*x*_) components of the spin–orbit curves of SO over nuclear geometries where the former couple states of same values of *Λ* (projection of the electronic angular momentum) and the latter couple states of different *Λ*. We see again that the effect of diabatisation is to smooth out the curves over *R*, where avoided crossings in the adiabatic picture create steep gradients around the avoided crossing. An example of the diabatisation process can be seen for the 〈e^1^Π|SO_*x*_|X^3^Σ^−^〉 and 〈(3)^1^Π|SO_*x*_|X^3^Σ^−^〉 pair in Fig. 10. Spin–orbit matrix elements at the internuclear separation of the PEC crossings are important in determining the possible spin–orbit induced predissociation mechanisms that occur between states of different spin multiplicity^26^ (see Discussion below). Referring to the spin–orbit couplings 〈e^1^Π|SO_*x*_|B^3^Σ^−^〉 and 〈d^1^Π|SO_*x*_|B^3^Σ^−^〉 in Fig. 3 with the magnitudes of approximately 90 and 20–30 cm^−1^, respectively, the predissociation of the B^3^Σ^−^ state through d^1^Π is likely to be very weak, but will be stronger through the e^1^Π state. The construction of diabatic SOCs will hence influence the efficiency of pre-dissociation pathways between states of different symmetry (Table 3).

**Table 3:** *Σ* values (total spin angular momentum projection onto the internuclear axis) for the bra and ket electronic states of the SOCs presented in Fig. 2 and 3

SO coupling	bra *Σ*	ket *Σ*	SO coupling	bra *Σ*	ket *Σ*	SO coupling	bra Σ	ket Σ
〈A^3^Π|SO_*x*_|X^3^Σ^−^〉	0	1	〈e^1^Π|SO_*x*_|X^3^Σ^−^〉	0	1	〈b^1^Σ^+^|SO_*z*_|B^3^Σ^−^〉	0	0
〈A^3^Π|SO_*x*_|A′^3^Δ〉	0	1	〈e^1^Π|SO_*x*_|B^3^Σ^−^〉	0	1	〈A′^3^Δ|SO_*z*_|A′^3^Δ〉	1	1
〈C^3^Π|SO_*x*_|A′^3^Δ〉	0	1	〈C^3^Π|SO_*x*_|A′′^3^Σ^+^〉	0	1	〈A′′^3^Σ^+^|SO_*z*_|X^3^Σ^−^〉	1	1
〈A^3^Π|SO_*x*_|B^3^Σ^−^〉	0	1	〈e^1^Π|SO_*x*_|A′^3^Δ〉	0	1	〈C^3^Π|SO_*z*_|A^3^Π〉	1	1
〈C^3^Π|SO_*x*_|X^3^Σ^−^〉	0	1	〈d^1^Π|SO_*x*_|X^3^Σ^−^〉	0	1	〈A^3^Π|SO_*z*_|A^3^Π〉	1	1
〈C^3^Π|SO_*x*_|B^3^Σ^−^〉	0	1	〈d^1^Π|SO_*x*_|B^3^Σ^−^〉	0	1	〈A′′^3^Σ^+^|SO_*z*_|B^3^Σ^−^〉	1	1
〈A^3^Π|SO_*x*_|A′′^3^Σ^+^〉	0	1	〈b^1^Σ^+^|SO_*z*_|X^3^Σ^−^〉	0	0	〈C^3^Π|SO_*z*_|C^3^Π〉	1	1
〈d^1^Π|SO_*x*_|A′^3^Δ〉	0	1	〈a^1^Δ|SO_*z*_|A′^3^Δ〉	0	0			

#### 3.2.3 Dipole moment curves

Fig. 4 and 5 plot the *z*- and *y*-components of the dipole moments coupling states of same and different symmetry (*Λ* plus multiplicity) respectively. The corresponding *μ*_*x*_ components can be always reconstructed from *μ*_*y*_ using their symmetry properties. We see again that the effect of diabatisation is to smooth out the curves over *R*, where now the DMCs tend to zero in the long range limit with no steep gradients caused by avoided crossings.

The vibronic intensities are directly affected by the derivatives of the dipole moment with respect to the internucelar separation, *R*. Since adiabatic curves are prone to strong, steep-gradient variations around avoided crossings, even small inaccuracies in *ab initio* calculations (including the position of the crossing and the corresponding NAC) can lead to large errors in spectral properties of the molecule. For example, the adiabatic 〈C^3^Π|DM|X^3^Σ^−^〉 dipole moment has a steep gradient at around 2 Å which can be expected to be due to the avoiding crossing between C^3^Π and C′^3^Π states, therefore the C^3^Π–X^3^Σ^−^ electronic band is expected to be sensitive to the quality of its adiabatic description. The diabatic representation can also be sensitive to the quality of the corresponding curves, but to a significantly lesser extend due to their smooth character.

Comparison with the 〈A^3^Π|*μ*_*x*_|X^3^Σ^−^〉, 〈C^3^Π|*μ*_*x*_|X^3^Σ^−^〉, and 〈B^3^Σ^−^|*μ*_*z*_|X^3^Σ^−^〉 transition dipoles provided by Sarka and Nanbu^27^ shows excellent agreements up to dissociation, with values ({ours, Sarka and Nanbu^27^}) at the ground state equilibrium geometry *R*_e_(X^3^Σ^−^) = 1.48 Å of {0.16,0.18} D, {0.333,0.337} D, {1.623,1.633} D, respectively.

### 3.3 Nuclear motion calculations

Duo^63^ is a general purpose variational (open access‡‡https://github.com/Exomol/Duo)github.com/Exomol.) code that solves the rovibronic Schrödinger equation for diatomics while allowing an arbitrary number of couplings between various electronic states including spin-spin, spin–orbit, spin-rotation, and rotational Born–Oppenheimer breakdown curves. It is assumed one has solved the Schrödinger equation for the electronic motion *a priori* in order to obtain PECs, SOCs, EAMCs, (T)DMCs *etc.* for the electronic states in question. These curves can be supplied to the program as either a grid of *ab initio* points, or in an analytical form. After solving the Schrödinger equation for the nuclear motion Duo obtains eigenstates and energies for the good quantum numbers *J* (total angular momentum), and *τ* (parity); other quantum numbers are assigned on the basis of the largest coefficient in the basis set. The eigenfunctions are used to compute transition line strengths and Einstein *A* coefficients in order to obtain a complete spectroscopy for the system in question. A detailed methodology of Duo is given by Duo.

### 3.4 The *ab initio* SO spectrum

Using the diabatic spectroscopic model we produce an *ab initio* rovibronic spectrum of SO for the system involving the lowest 11 singlet and triplet electronic states of SO covering the wavelength range up to 147 nm. The ‘active’ couplings within the spectrosocpic model used for cross-section calculations include 23 SOCs, 23 (transition) DMCs, and 14 EAMCs in-line with the couplings shown in Fig. 2–6. The vibrational sinc-DVR basis set was defined for a grid of 701 internuclear geometries in the range 0.6–6.0 Å. We select 58, 58, 49, 11, 31, 41, 27, 27, 14, 20, and 36 vibrational wavefunctions for the X^3^Σ^−^, a^1^Δ, b^1^Σ^+^, A^3^Π, B^3^Σ^−^, c^1^Σ^−^, A′′^3^Σ^+^, A′^3^Δ, C^3^Π, d^1^Π, and e^1^Π, respectively, to form the contracted vibronic basis. In total 15 364 624 Einstein A coefficients between 119 600 bound rovibronic states were computed with a maximum total rotational quantum number *J*_max_ = 180 and used to simulate rovibronic absorption spectra at a given temperature using the program ExoCross.^64^

**Fig. 11:**
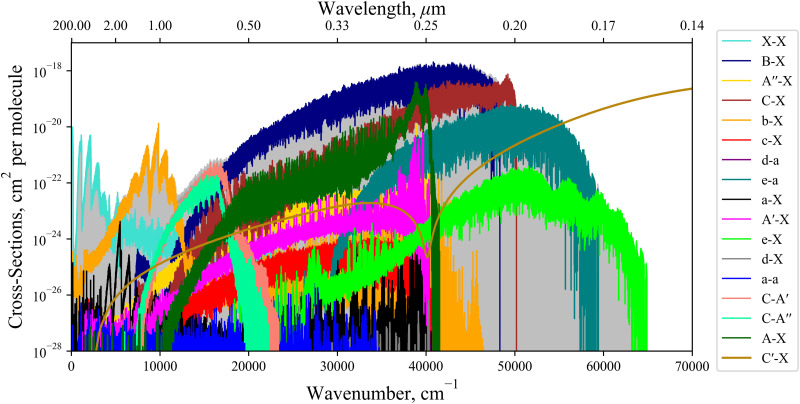
Dipole allowed and forbidden components of the *ab initio* absorption spectrum simulated with the diabatic model at 5000 K connecting the X^3^Σ^−^, a^1^Δ, A′^3^Δ, and A′′^3^Σ^+^ states. The C′^3^Π continuum is also plotted in gold. The absorption lines are modelled using Gaussian profiles with a HWHM of 0.6 cm^−1^ in the bound cases, and 300 cm^−1^ for the continuum band.

Fig. 11 shows an absorption rovibronic spectrum of SO computed at 5000 K with all bands plotted using different colours, both electric dipole allowed and forbidden. We model the spectrum at a high temperature for a visual aid since at this temperature there is a good separation between different electronic bands. The grey shaded region in Fig. 11 marks the total SO bound-bound absorption at 5000 K, which is mostly traced by the strongest bands with the exception for the region between 12 000–17 000 cm^−1^. Hence, weaker bands, *e.g.* ones that break dipole selection rules, have negligible contribution to the total SO opacity and will be less important for low resolution studies such as in astrophysical observations.

The non-bound diabatic states such as C′^3^Π and (3)^1^Π are excluded from the bound-bound spectra simulations. Our tests show that the effect of the unbound states on the bound-bound spectra is negligible, and *vice versa*, the continuum spectra are negligibly affected by the bound electronic states and therefore can be treated separately.

The continuum spectra of the non-bound diabatic C′^3^Π state is computed using the method described in Pezzella *et al.*^65^ and is shown in Fig. 11, plotted in gold and overlaying the bound-bound spectrum to demonstrate its contribution to the total SO opacity. For the continuum state a larger basis set of 5000 wavefunctions was used. The structure energetically above (below) the ‘dip’ at 41 200 cm^−1^ is due to absorption to unbound C′^3^Π states above (below) the S(^1^D) +O(^3^P) dissociation. The X^3^Σ^−^ → C′^3^Π continuum band continues to 100 000 cm^−1^, peaking at ∼78 000 cm^−1^ which corresponds to the most vertical transitions from states localised around the minima of X^3^Σ^−^.

We note that the dipole-forbidden bands in Fig. 11 are not computed using quadrupole or magnetic dipole moments, which have very weak intensities, but rather intensities are ‘stolen’ from other transitions. This intensity stealing propagates through the mixture of electronic wave-functions *via* couplings such as SOCs and EAMCs. For example, the spin-forbidden c^1^Σ^−^–X^3^Σ^−^ band occurs due to the overlap between the c^1^Σ^−^ wavefunction both with e^1^Π and d^1^Π wavefunctions through the EAM couplings, and then with X^3^Σ^−^ through a secondary mixing *via* 〈e^1^Π|SO_*X*_|X^3^Σ^−^〉 and 〈d^1^Π|SO_*X*_|X^3^Σ^−^〉 to produce a direct dipole moment, which dominates over the weaker magnetic and quadrupole moment mechanisms.

We compute absolute intensities for every rovibronic transitions between the lowest 11 diabatic singlet and triplet states of SO covering the entire spectroscopic range up to 147 nm, where NACs are treated. We note that the only other study with similar coverage into the UV on SO is from the theoretical work by Sarka and Nanbu^27^ who compute cross sections for 190–300 nm. However, our spectroscopic model is both more complete and phase consistent (phases carefully reconstructed, see Section 3.2.2), whereas Sarka and Nanbu^27^ do not provide any phases.

### 3.5 Experimental coverage of the *ab initio* SO spectrum

**Fig. 12:**
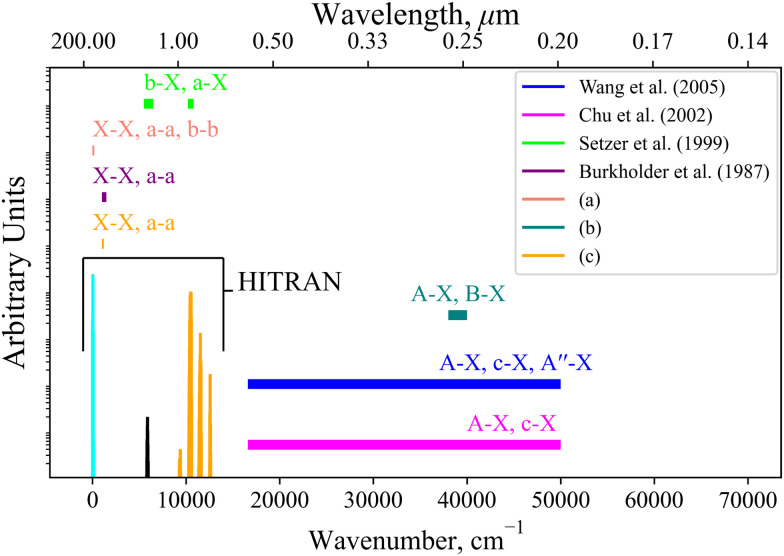
Coverage of experimental measurements for 24 sources illustrated by horizontal bars covering the spectral regions measured, where the named works^6,18,66,67^ include some spectral data, mostly with relative intensities: (a) 14 sources^10,14,15,30,68–78^ cover X^3^Σ^−^ → X^3^Σ^−^, a^1^Δ → a^1^Δ, b^1^Σ^+^ → b^1^Σ^+^ for 0–125 cm^−1^; (b) 3 experimental sources^17,19,20^ cover the A^3^Π → X^3^Σ^−^ and B^3^Σ^−^ → X^3^Σ^−^ bands for 38 000–39 800 cm^−1^; (c) 2 experimental sources^21,23^ cover the X^3^Σ^−^ → X^3^Σ^−^ and a^1^Δ → a^1^Δ bands for 1040–1125 cm^−1^.

Currently within the literature a small fraction of the SO spectrum has been measured experimentally covering only the X^3^Σ^−^, a^1^Δ, b^1^Σ^+^, A^3^Π, and B^3^Σ^−^ states. Fig. 12 reviews the spectroscopic coverage of SO from 24 experimental sources from the literature. Fig. 11 shows our model to supplement the experimental data over the whole spectral range. In particular, we cover the SO spectrum above 40 000 cm^−1^ and 12 000–16 000 cm^−1^ where no measurements have been taken for any electronic state. We also plot the available Hitran^28^ SO line list containing data on the first three electronic states X^3^Σ^−^, a^1^Δ, and b^1^Σ^+^. Our *ab initio* model is able to extend the Hitran coverage up to dissociation at 40 000 cm^−1^.

The aim of a future work is to refine our *ab initio* SO model to the experimental transition frequencies from these sources and to produce an empirically accurate line list for SO.

## 4 Effect of diabatisation on the computed spectra

In theory, the adiabatic and diabatic representations should provide identical results, provided that the corresponding NACs and DCs are included. However, due to the computational cost of computing NACs through proper *ab initio* methods, it is not common practice to include NACs in adiabatic models. Without definition of the NAC, non-Born–Oppenheimer interactions are effectively removed.

In this section we analyse the importance of the non-adiabatic couplings between C^3^Π and C′^3^Π as well as between e^1^Π and (3)^1^Π for computing the (absorption) spectra of SO. To this end we consider our diabatic spectroscopic model to be complete and use it to compute a reference absorption spectrum of SO. This spectrum is then used to compare with an ‘adiabatic’ spectrum of SO computed using the non-adiabatic curves without NACs.

**Fig. 13:**
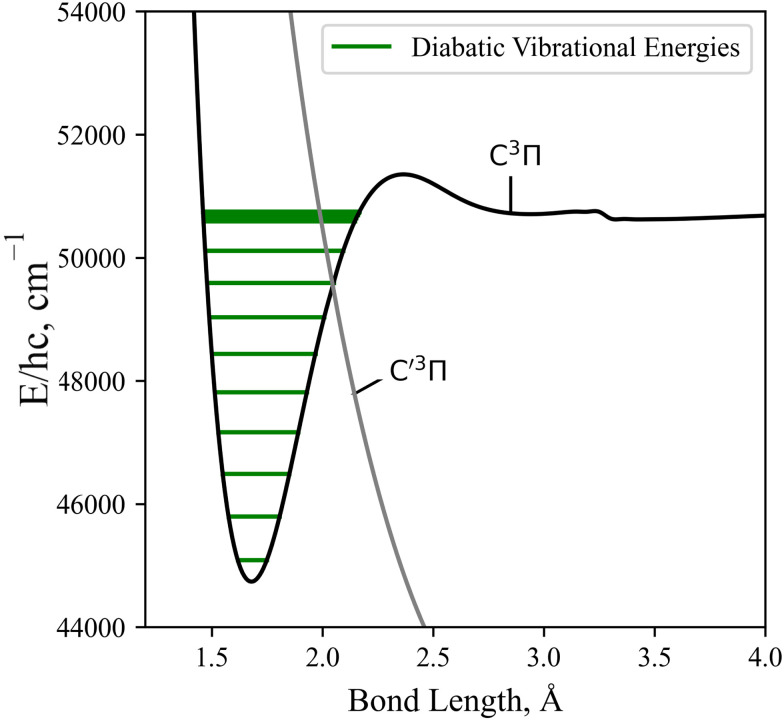
The computed Duo diabatic vibronic energies of the C^3^Π superimposed upon the C^3^Π and C′^3^Π PECs. The secondary and tertiary ‘bumps’ at *R* = 2.42, 3.30 Å are due to an avoided crossing and numerical noise, respectively.

As detailed in Section 3.1, the diabatisation of the potential energy curves induces non-zero off-diagonal elements *via* corresponding 2 × 2 potential energy matrices in the diabatic representation. These off-diagonal coupling elements will be referred to as diabatic couplings, DCs, which are characterised by cusp shaped curves centered at the avoided crossing. As before, in the diabatic spectrum simulations the unbound states are excluded. Fig. 13 illustrates the vibrational energy levels of the diabatic C^3^Π, fully bound below its dissociation limit of 50 700 cm^−1^.

**Fig. 14:**
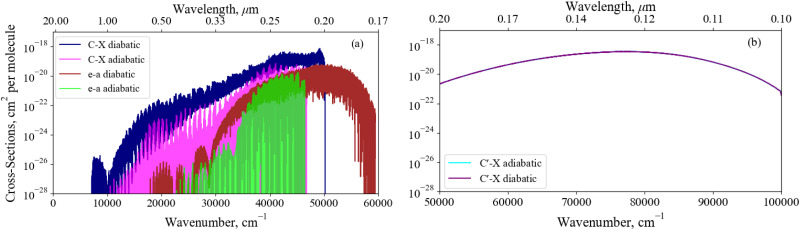
A comparison between the X^3^Σ^−^ → C^3^Π, a^1^Δ → e^1^Π, and X^3^Σ^−^ → C′^3^Π band spectra computed with an adiabatic model with no defined NAC and a diabatic model. These bands are dipole allowed and are expected to be observable, we see great differences between the spectra at the dissociation, highlighting the importance of diabatisation. Each spectra has been modelled at a temperature of 5000 K with Guassian line profiles of a 0.6 cm^−1^ HWHM.

Fig. 14 illustrates the importance of the non-adiabatic effects when modeling the spectra around avoiding crossings for the X^3^Σ^−^ → C^3^Π and a^1^Δ → e′Π bound-bound absorption bands (panel a), and the X^3^Σ^−^ → C′^3^Π continuum absorption band (panel b). The adiabatic spectra were computed with the NACs excluded and compared to the diabatic spectra with the non-adiabatic effects fully encountered. Each spectra has been modelled at a temperature of 5000 K – such that hot bands are populated, aiding our comparisons below – with Gaussian profiles of a 0.6 cm^−1^ half-width-at-half-maximum (HWHM) for the bound-bound spectra and a HWHM of 300 cm^−1^ for continuum bands.

Great differences between the bound-bound spectra in panel (a) of Fig. 14 are seen towards both the high and low energy regions. In the high energy region the adiabatic spectral bands terminate abruptly at the avoided crossings whereas the diabatic bands continue to the diabatically correlated dissociation asymptotes S(^1^D) + O(^3^P) & S(^1^D) + O(^1^D) (see Fig. 1). The diabatisation extends these bands by at least a few thousand wavenumbers because of the availability of higher rovibrational states in the deeper diabatic potential wells. For purely bound-bound calculations, the adiabatically computed bands have lower intensities compared to the diabatic spectrum which can be attributed to the increased repulsive character of the adiabatic PECs on the right hand side of the crossing points present. Due to the tunneling through the potential barriers, the adiabatic wavefunctions ‘leak’ to the continuum region thus resulting in reduction of the intensity of their bound absorption spectra. The most interesting feature from Fig. 14 is the extension of the a^1^Δ → e^1^Π band beyond the stronger X^3^Σ^−^ → C^3^Π band at *E*/*hc* > 50 000 cm^−1^. Although being relatively weak, this band is not covered by stronger bands and therefore may be observable in the ∼0.18–0.2 μm region, a result only predicted when using a full non-adiabatic theoretical treatment.

The low intensity regions are very sensitive to changes in the *ab initio* model and will be also affected by the changes in the shape of couplings between the adiabatic and diabatic representations. The hump-like structure in the C^3^Π–X^3^Σ^−^ band at around 10 000 cm^−1^ is absent in the adiabatic spectrum because of the unavailability of vibrational states above the avoided crossing. For example, the brightest transitions within this hump for the C^3^Π–X^3^Σ^−^ band connect the *ν* = 13 state which is energetically above the avoided crossing in the adiabatic PEC.

We note that these regions negligibly contribute to the total SO opacity and so are not important for the SO model, but will be important for other systems where non-adiabatic effects occur in the spectroscopically important regions.

Panel (b) of Fig. 14 presents a similar analysis for the continuum X^3^Σ^−^ → C′^3^Π band, which would include an additional bound structure towards longer wavelengths if the NAC is not included in the adiabatic model since the C′^3^Π PEC in this representation is bound. However, the adiabatic X^3^Σ^−^ → C^3^Π bound feature is orders of magnitude weaker than the continuum bands presented here and an analysis on the change of character of bound-bound absorption bands with diabatisation is already provided above. The X^3^Σ^−^ → C′^3^Π continuum bands for transitions to unbound C′^3^Π states above the S(^1^D) +O(^3^P) dissociation converge between both representations, since the non-adiabatic effects are far away from the peak at ∼78 000 cm^−1^ corresponding to vertical transitions from the electronic ground state. However, if the avoided crossing occurred vertically above the X^3^Σ^−^ minima, we would expect non-adiabtaic effects to have a greater contribution to the continuum cross sections.

From the comparison above, we show that neglecting NACs within an adiabatic model can lead to drastic differences in the physics gleamed from the computed spectra.

## 5 Conclusions

In this work, we use multireference methods of electronic structure theory combined with a diabatisation procedure to compute a fully diabatic model for the transient diatomic molecule sulphur monoxide. The model includes 23 spin–orbit, 23 (transition) dipole moment, and 14 electronic angular momentum curves for the X^3^Σ^−^, a^1^Δ, b^1^Σ^+^, A^3^Π, B^3^Σ^−^, c^1^Σ^−^, A′′^3^Σ^+^, A′^3^Δ, C^3^Π, d^1^Π, and e^1^Π electronic states of SO and were produced *ab initio via* CASSCF and MRCI methods using aug-cc-pV5Z basis sets. These curves were then used to compute the nuclear motion *via* solving the fully-coupled Schrödinger equation with the Duo program. A further two electronic states (C′^3^Π and (3)^1^Π) were computed along with their couplings, which are essential to forming the diabatic representation. The diabatisation procedure we present is a computationally low cost method to reconstruct the non-adiabatic couplings *a priori* to nuclear motion calculations. To assess the importance of non-adiabatic effects for the spectroscopy of SO, we compare spectra computed in the diabatic and adiabatic representations without definition of NACs. The most notable difference is the absence of the UV spectrum above ∼50 000 cm^−1^ because of the illusionary predissociation from the adiabatic PECs. We also saw the adiabatically computed bound absorption bands to have lower intensities than the diabatic counterparts. It is therefore important to treat NACs for systems where these non-adiabatic interactions occur in spectroscopically important regions since they have drastic effects on spectral structure.

All coupling curves of SO are defined with self-consistent relative phases, which is crucial for spectral calculations.^31^ Therefore our spectroscopic model of SO provides a comprehensive and extensive theoretical baseline, which is the first fully reproducible spectroscopic description of SO longer than 147 nm. Since the existing spectroscopic data on SO only covers X^3^Σ^−^, a^1^Δ, b^1^Σ^+^, A^3^Π, and B^3^Σ^−^, our *ab initio* model can be used as a benchmark for future rovibronic methods and calculations.

The topic of our next work will be to build a semi-empirical line list as part of the ExoMol project^79,80^ for SO through the refinement of our *ab initio* model to experimental transition data, where we expect to reduce the shift in line positions relative to experiment. The final SO line list will have applications primarily in the atmospheric modelling of exoplanets^81–83^ and cool stars. Further applications of this empirical SO line list will be in shock zone modelling,^11,12,84^ SO lasing systems,^7,8^ and spectroscopy of Venus^85,86^ and Io.^87,88^

## Data availability

The *ab initio* curves of SO obtained in this study are phase consistent and are provided as part of the supplementary material to this paper along with our spectroscopic model in a form of a Duo input file. The line list computed with Duo can be directly used with the ExoCross program to simulate the spectral properties of SO. We also provide a Julia source code used for the diabatisation procedure is provided as part of the ESI.†

## Conflicts of interest

There are no conflicts to declare.

## Supplementary Material

CP-024-D2CP03051A-s001

## Appendices

### Appendix

#### A.1 The Geometric average of DCs

We show here the simple geometric average used to combine the DCs arising from our NACs modelled with a Lorentzian and a Laplacian. We have from eqn (16) of An and Baeck^48^ the followingwhere in the second line the matrix elements given in eqn (4) are substituted. Comparing the last two lines, one can recover eqn (8), the mixing angle that corresponds to the geometrically averaged DC is thenwhich corresponds to eqn (15) of An and Baeck.^48^

#### A.2 Diabatisation procedure

Non-adiabatic interactions cause steep gradients around the region of the avoided crossings in both PECs and coupling curves. To diabatise these couplings one requires the operation of the unitary transformation matrix given in eqn (1). For some coupling, *ζ*, and considering two adiabatically interacting states |1〉 and |2〉, the exact transformation for non-diagonal couplings 〈1/2|*ζ*|*k*〉, where *k* ≠ 1, 2, from the adiabatic (‘a’) to the diabatic (‘d’) representation can be found as followsHowever, diagonal couplings also require the off-diagonal counterparts, and transform similarly to the potential energies as,

The computational procedure for performing the diabatisation is straightforward, and is based on the heuristic that the diabatisation should result in continuous potential energy curves that are twice differentiable due to the properties of the wave function and derivatives. For a function represented by a discrete grid of points *V*_*i*_ at geometries *R*_*i*_, gradients are calculated *via* finite differences and the task of satisfying this criterion is approximated by minimizing the sum of second derivatives of the function.

To achieve this we optimize the parameters of an arbitrary NAC function, *ϕ*(*R*;{*p*}). The parameters {*p*} are typically: a central geometry *R*_c_, and a characteristic width *ω* (see *e.g.*eqn (5) and (6)). The NAC function, in turn, parameterises the transformation from the adiabatic potential energy operators ***V***^a^_*i*_ to the diabatic potential energy operators ***V***^d^_*i*_ at each geometry *i*, as described by eqn (4).

The optimization itself is easy to achieve using any commonly available optimization libraries. In the present work we use the Julia programming language and the open-source Optim library's Optim.minimizer function to minimize the following loss function using the Nelder–Mead method:^89,90^where the terms of the summation are simply central finite difference second derivatives of the diabatic potential energy operator.

The integration of the NAC function to obtain the mixing angle *β*_*i*_ at each geometry is obtained by adaptive Gauss-Kronrod quadrature using the QuadGK.jl library.

Empirically we find that an initial guess for the characteristic width *ω* with the correct order of magnitude suffices for the optimizer to converge. However, the procedure is somewhat more sensitive to the initial guess for the central geometry *R*_c_ as a result we provide a convenience function that attempts to detect the central geometry by searching for the largest absolute value of the second order derivatives of the adiabatic operators. The source code for the diabatisation procedure is provided as part of the ESI.†
